# Everything robots need to know about cooking actions: creating actionable knowledge graphs to support robotic meal preparation

**DOI:** 10.3389/frobt.2025.1682031

**Published:** 2025-10-29

**Authors:** Michaela Kümpel, Manuel Scheibl, Jan-Philipp Töberg, Vanessa Hassouna, Philipp Cimiano, Britta Wrede, Michael Beetz

**Affiliations:** 1 Institute for Artificial Intelligence, Bremen University, Bremen, Germany; 2 Medical Assistance Systems Group, Medical School OWL, Bielefeld University, Bielefeld, Germany; 3 Center for Cognitive Interaction Technology, Bielefeld University, Bielefeld, Germany

**Keywords:** robot manipulation, knowledge graph, recipe analysis, meal preparation, large language models

## Abstract

This paper addresses the challenge of enabling robots to autonomously prepare meals by bridging natural language recipe instructions and robotic action execution. We propose a novel methodology leveraging Actionable Knowledge Graphs to map recipe instructions into six core categories of robotic manipulation tasks, termed Action Cores cutting, pouring, mixing, preparing, pick and place, and cook and cool. Each AC is subdivided into Action Groups which represent a specific motion parameterization required for task execution. Using the Recipe1M + dataset (Marín et al., IEEE Transactions on Pattern Analysis and Machine Intelligence, 2021, 43, 187–203), encompassing over one million recipes, we systematically analysed action verbs and matched them to ACs by using direct matching and cosine similarity, achieving a coverage of 76.5%. For the unmatched verbs, we employ a neuro-symbolic approach, matching verbs to existing AGs or generating new action cores utilizing a Large Language Model Our findings highlight the versatility of AKGs in adapting general plans to specific robotic tasks, validated through an experimental application in a meal preparation scenario. This work sets a foundation for adaptive robotic systems capable of performing a wide array of complex culinary tasks with minimal human intervention.

## 1 Introduction

Robots (still) do not prepare our daily dishes, since the manipulation skills involved in meal preparation actions are very complex. Even if we consider only a single action category like cutting, we have to account for many factors that influence the execution and the desired goal state, such as object properties (e.g., the existence of a peel), task variations (such as halving or slicing) and their influence on motion parameters, as well as the situational context (e.g., the available tools).

To successfully compute the body motions needed to execute different recipe instructions, robots need *knowledge*. This work addresses the question how we can build knowledge bases for meal preparation actions that robots can use to translate the contained information into body motion parameters.

While recent research has focused on translating natural language instructions to parameters for pick and place tasks (see Ahn et al., 2023; Rana et al., 2023), preparation of recipes requires more knowledge than grounded environment information. Recipes contain:

1. Commonsense knowledge such as that a cup sometimes is used as a container but sometimes as a measurement unit
2. Environment and physics knowledge such as what objects can be used for the task and that a filled cup should be held upright
3. Action and manipulation knowledge such as how a task is broken down into motor primitives but also that hot content might burn your finger/gripper, so a hot cup should be held by its handle

Thus, accomplishing complex manipulation tasks for the preparation of recipes can be stated as a reasoning problem: Given a list of vague task requests such as *“Set aside for 15 min, then drain and put into a blender”*, infer the objects to use based on the text and the current scene graph of the environment, as well as the necessary body motions to achieve the desired result while avoiding unwanted side effects. The main question is: How can we build knowledge bases that represent this knowledge in a machine-understandable way?

Kümpel (2024) proposes a methodology to create *Actionable Knowledge Graphs* as knowledge bases that robots can use for action execution. An *Actionable Knowledge Graph* (AKG) connects object information to environment information and action information for an embodiment of knowledge (Kümpel, 2025). AKGs provide action parameters for different Action Groups (AGs) of an action category, which can be used in general action plans (Hassouna et al., 2024) for the execution of task variations such as for performing slicing, dicing and halving derived from a general action plan for cutting (Kümpel et al., 2024; Beetz et al., 2024). Hence, AGs provide agents with knowledge about how a particular activity shall be performed in a specific context, along with the awareness about objects involved in these actions as well as the properties that influence task execution. For the example use case of cutting fruits, such an AKG has been used by a robot to infer the necessary body motions for a range of cutting tasks on different objects, from slicing a cucumber to halving an apple (Beetz et al., 2024; Kümpel and Töberg, 2024).

Still, in order for a robot to prepare any recipe given the enormous - and possibly open ended - amount of recipes, the question has to be asked if it is possible to derive such AKGs for all recipes. We divide this question into the following sub-questions:

1. How many action verbs, and corresponding groups, occur in recipes?
2. How do we structure AKGs to cover these verbs and groups?

**FIGURE 1:**
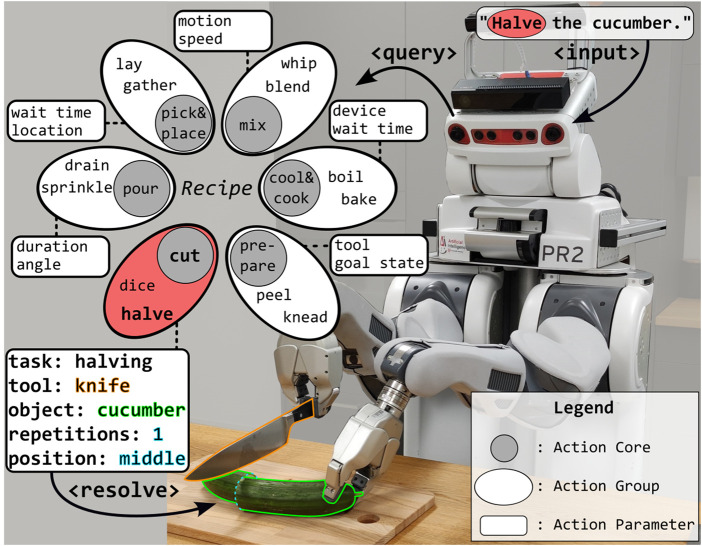
Motivation of this work: using Action Cores and Action Groups to parametrise generalised action plans.

**FIGURE 2:**
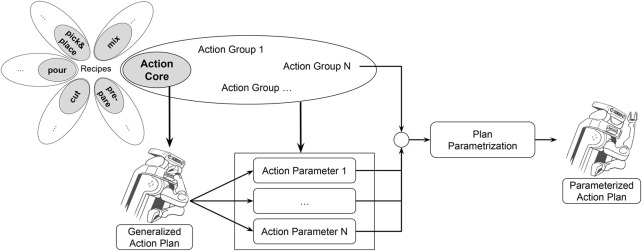
Connection between action cores (AC), action groups (AG) and the manipulation plan employed by the robot.

This paper answers these questions by using Action Cores (ACs) (an AC is a main manipulation capability like cutting that can be translated to a general action plan), Action Groups (AGs) (an AC consists of several more specific AGs that use a similar manipulation plan and thus result in similar body movements and outputs, e.g., the AC of cutting consists of the AGs dicing, slicing, etc.) as well as an Actionable Knowledge Graph, that contains task, object and environment knowledge and enables robots to infer the body motions needed to prepare any given recipe. We visualize the connection between these concepts in Figure 1 and in Figure 2.

We hypothesize that recipes consist of six main ACs that can be broken down into several AGs, as visualized in Figure 1. To test this hypothesis, we created AKGs for these six main ACs, analysed the *Recipe1M + corpus* (Marín et al., 2021) consisting of 1,028,692 recipes for the occurring instructional verbs and matched them with the AGs of the AKGs.

**FIGURE 3:**
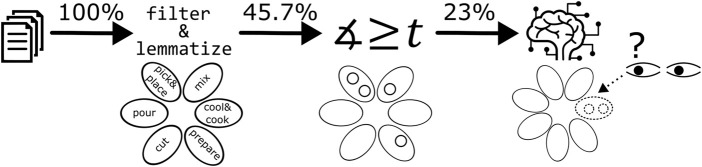
From the recipe corpus, all included verbs are being matched against the Action Cores and Groups in three steps.

In a first matching step where only direct matches between lemmatized and prefix-trimmed verbs from the recipes and actions in our proposed ACs were considered, we found that the six proposed ACs cover roughly 54% of actions in the corpus. To extend these results, we employed cosine similarity to match all actions above a certain, experimentally defined threshold, bringing the coverage up to ∼77% . For the remaining unmatched verbs, we employed a neuro-symbolic approach, matching verbs to existing AGs or generating new action categories by employing a set of Large Language Models (LLMs). The complete pipeline is visualized in Figure 3.

The contributions of this paper are the following:

- We define the six main Action Cores for meal preparation tasks.
- We create Actionable Knowledge Graphs for the ACs.
- We perform a neuro-symbolic experiment to match verbs of recipe instructions with our AKGs and categorise verbs that are not covered by our AKGs.

The contributions are validated by letting various simulated robots execute multiple tasks, in different environments^1^. We also created an interactive website where users can choose a recipe and get the list of matched actions with their respective body motion parameters^2^.

## 2 Related work

Correctly executing unknown tasks is still a major challenge in robotics due to the fact that tasks are often underspecified and assume commonsense knowledge about objects and the environment (Töberg et al., 2024). Previous approaches like the work by Forbes and Choi (2017) try to infer the implicitly embedded physical knowledge centred around actions and their participating objects. However, for the execution of unknown meal preparation tasks, physical implications focused on size or weight are not conclusive enough to empower robots.

For meal preparation tasks, recipes usually offer preparation instructions, which are structured task sequences written in natural language. To support robotic execution as well as general learning tasks, previous work has focused on analysing different aspects of recipes. For example, Yasukawa and Scholer (2017) focus on the concurrency of dish titles and ingredients whereas the work by Nyga and Beetz (2012) analyses actions and their frequency in a WikiHow corpus, an approach we adopt and adapt in this work. Another work by Kiddon et al. (2015) focuses on mapping recipe instructions to action graphs describing the participating objects and the order of instructed actions, an important aspect that we also integrate in this work.

However, before a robot can successfully prepare a meal, it needs a proper understanding of recipes and their instructions, which is demonstrated by the benchmark introduced by Nevens et al. (2024). As a first step, the procedural text can be transformed into more structured representations like LTL formulae (Mavrogiannis et al., 2024) or functional networks (Paulius et al., 2024) by, e.g., employing the language processing capabilities of LLMs. This preprocessing also allows for additional semantic annotations that can support the later action execution, e.g., by adding the in- and output objects to each step in the recipe instructions (Diallo et al., 2024). For the correct execution, robots can rely on symbolic planning (Bollini et al., 2013), functional networks combined with task trees (Sakib et al., 2022; Sakib and Sun, 2024), large and vision language models (Kanazawa et al., 2024; Paulius et al., 2024) or human demonstrations (Scheutz et al., 2025). In works like (Siburian et al., 2025), the domain-specific skills relevant for food preparation are added on top of an integrated task and motion planning framework to allow the robotic agent to perform force-based tip detection or reinforcement learning-based slicing. While all these works have brought us closer to deploying kitchen robots, they usually rely on previously defined task knowledge and miss an important aspect in meal preparation: flexible translation of specific task variations into diverse body motions - i.e., being able to differentiate between slicing and dicing and how this affects body motions.

In our approach, we create action cores and groups and their corresponding knowledge graphs to provide the robot’s cognitive architecture with access to situationally relevant knowledge as a basis to parametrise generalised action plans. Our created action representation is hierarchical and similar to the hierarchical action taxonomy by Pereira et al. (2022), which focuses on actions performed by service workers in the food industry. However, their work envisions different machines and robots for executing the different actions, whereas we empower a single robot to execute all actions in our cores.

To also include actions not covered by our six action cores, we conduct an experiment with a neuro-symbolic approach for classification. Using a LLM to automatically categorise new entities into unknown classes is also proposed by Høeg and Tingelstad (2022). Generally, the topic of automatic sorting has been explored by, e.g., Guérin et al. (2018), but in most cases this problem is focused on objects instead of actions. We do not employ the LLMs to directly generate manipulation plans, since previous work has shown them to be ineffective for generating plans for complex cognitive architectures (Töberg et al., 2025).

## 3 From recipes to body motions

This work is based on prior work on analysing the amount of different actions occurring in the WikiHow corpus (Nyga and Beetz, 2012), where the authors found that the top 15 action verbs occur in more than 50% of instructions in WikiHow recipes. We go a step further and hypothesize that most action verbs occurring in recipe instructions can actually be broken down into six main ACs that contain AGs. The set of 6 ACs was derived through an iterative, empirically grounded process. We began with frequency analysis in Section 3.1 of more than 21M verbs in the *Recipe1M*+ (Marín et al., 2021) corpus, as can be seen in Table 1, which revealed clusters of high-frequency action families (e.g., cut, mix, pour). The Recipe1M + dataset contains 1,028,692 recipes with 10,767,598 instructions collected from different sources and written in natural language^3^. Each recipe also contains an ingredient list and associated food images, but in this work we focus on analysing the verbs that occur in the preparation instructions. The action clusters found in the corpus suggest candidate manipulation primitives.

Considering the action verb frequencies, we propose to classify them into the six main ACs of *cutting, pouring, mixing, preparing, pick and place, cook and cool* - a compact yet expressive set of ACs - that comprise of several AGs, which can be translated to motion parameters of robot action plans, as will be explained in the following. As mentioned above, a visual summary of these concepts is depicted in Figure 2. We evaluated coverage of the created ACs against the corpus and found that introducing six categories leads to a high coverage.

### 3.1 Analysing action verb frequencies

**FIGURE 4:**
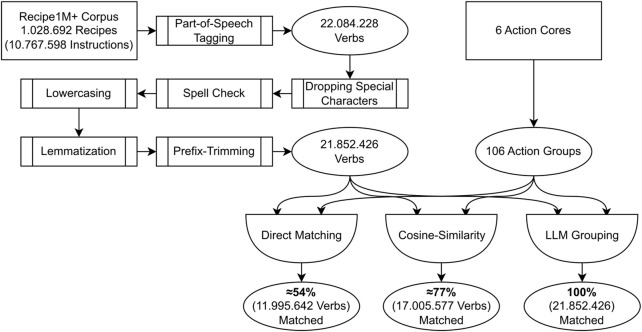
Flowchart that visualizes the process of preprocessing the database of recipes and of matching this body of verbs to the action groups of the action cores.

The 1,028,692 recipes from Recipe1M+ were used as the input data for the *Spacy* library to assess the dependency trees of the given recipes’ instructions. For the analysis we used the pre-trained en_core_web_trf model^4^, which is based on the RoBERTa architecture (Liu et al., 2019). The overall process of matching the verbs of the recipes to the ACs is visualized in Figure 4.

In a first step, the recipes and their instructions were parsed by extracting all words classified as verbs after part-of-speech tagging, resulting in 22,084,228 words. To further process these verbs, special characters were dropped, a spell check was applied and the verbs were all transformed into lowercase. The remaining verbs were lemmatized to bring them into their infinitive case. Additionally, we trim prefixes from the verbs in the corpus that only change their meaning in, e.g., a temporal, spatial, or negating fashion (prefixes such as *un-, re-, pre-, post-, …*). This preprocessing resulted in 21,852,426 verbs.

At this point the database of verbs still included duplicates, which are not necessary for further assessment. Still, a frequency check at this point provides valuable insights into the most prominent verbs of the recipe set. In Table 1 the 20 most frequent verbs in the corpus are listed. The fourth column shows the number of times that this particular verb (after pre-processing and lemmatization) is included in the set of recipes. The fifth column lists the relative frequency of a verb in the entire corpus. The 20 most common verbs make up for about 47.2% of all verbs.

**TABLE 1:** 20 most common verbs in the Recipe1M + corpus (Marín et al., 2021). They make up for about 47.2% of all 21,852,426 found verbs.

#	Verb	Action core	Frequency	Freq. [%]
1	add	mix	1572723	7.197
2	stir	mix	914195	4.183
3	cook	cook	827379	3.786
4	heat	cook	672788	3.079
5	serve	pick and place	599245	2.742
6	place	pick and place	568270	2.600
7	mix	mix	557993	2.553
8	cover	pick and place	476680	2.181
9	bake	cook	461082	2.110
10	move	pick and place	460446	2.107
11	combine	mix	453793	2.077
12	use	pick and place	365250	1.671
13	pour	pour	350040	1.601
14	cool	cool	318706	1.458
15	remain	-	310116	1.419
16	set	pick and place	297437	1.361
17	cut	cut	291629	1.335
18	make	-	287965	1.318
19	turn	pick and place	271807	1.244
20	sprinkle	pour	260417	1.192
∑		18/20 matched	10,317,961	47.217

### 3.2 Action cores

Looking at Table 1, we can identify six main Action Cores that consist of one hypernym for general preparation actions (prepare), three main manipulation actions (cutting, pouring, mixing) and two main categories for tool and device use (pick and place, cool and cook):

- Preparing: Many recipes include preparation tasks to prepare the food objects for further handling or bringing them into a desired shape. Many of these tasks (e.g., peeling, kneading) are difficult to be performed by a robot, unless they use a tool/device. Hence, we created this rather broad category that will be focus of future work.
- Pouring: Pouring is an action with a simple motion but where the specific task, object properties and ingredient consistency heavily influence motion parameters and successful action execution.
- Cutting: Cutting is an action with a complex motion sequence and the goal of dividing an object into two or more pieces of a certain shape. Its execution is influenced by the specific task and object properties.
- Mixing: Mixing can result in a range of different motions, some of which require certain tools or containers. Its execution depends on the specific task, available objects, as well as ingredient consistency and temperature.
- Pick and Place: Pick and Place tasks have been a research focus. Here, for most tasks the focus lies more on object properties that influence successful grasping or specific locations where the object should be placed. We differentiate between pick and place tasks, picking tasks (e.g., “take”), and placing tasks (e.g., “put”).
- Cook and Cool: Heating and cooling tasks make up an important AC in meal preparation, but can be broken down into Pick and Place tasks that involve a device (e.g., placing in an oven/microwave) and a device interaction task (e.g., turning the oven/microwave on).

The verb list in Table 1 shows two unmatched verbs: “remain” and “make”. We hypothesise that “remain” was falsely classified as a verb although it was used as an adverb in the recipe instructions (as in “add the remaining ingredients”). The word “make” is also not used as an instruction leading to an action but rather as an auxiliary verb (i.e., “To make the filling/cake, put … “). Thus, we do not consider these two as action verbs that should be included in the ACs.

From the perspective of robotic manipulation, the 6 ACs are also non-overlapping. Each AC corresponds to a distinct manipulation primitive as detailed in Table 2. These primitives require distinct motor skills and parameterizations, which makes them particularly suitable as a structured basis for robotic execution.

**TABLE 2:** Illustrative examples of verbs grouped into the 6 ACs and their correspondence to manipulation primitive.

Action core	Sample verbs	Manipulation primitive
Cutting	cut, halve, slice, dice	Partitioning an object into pieces of a certain shape/size
Mixing	mix, stir, whisk, blend	Homogenizing multiple ingredients into a mixture
Pouring	pour, drain, sprinkle	Transferring material via gravity or controlled flow
Pick and Place	put, place, move, serve	Relocating objects from one location to another
Cook and Cool	cook, bake, boil, freeze	Changing thermal state of ingredients using devices
Preparing	peel, knead, unpack	Transforming an ingredient for further handling

### 3.3 Action groups

Previous work by Kümpel et al. has proposed the creation of an Actionable Knowledge Graph for the example use case of cutting fruits (Kümpel, 2024; Kümpel et al., 2024). Amongst other things, this AKG acquires knowledge from different sources (Beetz et al., 2024), such as synonyms and hyponyms for “cutting” from WordNet (Miller, 1995), VerbNet (Schuler, 2005) and FrameNet (Baker et al., 1998). The authors propose to group these verbs into Action Groups of verbs that result in similar motion parameters, and output. As a result, verbs like chopping, mincing and cubing are assigned to the “dicing” AG, which results in different motion parameters than the AGs of halving, slicing, or cutting.

Since the concrete instantiation of parameters is the same for all actions in one AG, using them to cluster similar actions can simplify the actual execution and increase the coverage of novel actions. This makes AGs a crucial influence factor for robots being able to successfully infer and differentiate the body motions for a specific task. Therefore, we reuse the concept of AGs and create them for our 6 ACs. With this, the action verbs in our AKGs cover ∼54% of the verbs in the recipe corpus.

### 3.4 Actionable knowledge graph for cooking actions

To make the structure of the Actionable Knowledge Graph (AKG) more tangible, we provide a minimal fragment covering three representative Action Groups: Dicing (from the Cutting AC), Draining (from the Pouring AC), and Stirring (from the Mixing AC). Table 3 lists selected knowledge triples in RDF style (subject–predicate–object). Each triple encodes either a tool association, an input/output relation, or a motion parameterization required for robotic execution.

**TABLE 3:** Excerpt of the Actionable Knowledge Graph (AKG) showing triples for three action groups.

Subject	Predicate	Object
Dicing	requiresPriorTask	Julienning
Dicing	hasInputObject	Stripe
Dicing	hasResultObject	1 Cube ∧ 1 Stripe
Dicing	affordsPosition	SlicingPosition
Dicing	repetitions	n
Draining	hasParticipant	Seave
Broth	subClassOf	Food
Broth	hasConsistency	Liquid
PouringAngle45Degree	hasInputObject	Food
AND	hasConsistency	Liquid
PouringAngle45Degree	valueQuantity	min 1 ∧ max 45
Stirring	affordsTrigger	MixingTool
Stirring	hasInputObject	min 2 Food
Stirring	requiresMotion	OrbitalMotion
OrbitalMotion	radiusLowerBoundRelative	0.7
OrbitalMotion	radiusUpperBoundRelative	0.7

The parameters illustrated in Table 3 represent a sufficient and reliable basis for executing the corresponding manipulation actions, as they capture essential preconditions, input and output relations and motion constraints. At the same time, we emphasize that the representation is deliberately simplified: it abstracts away from the wide variety of possible execution strategies and object-specific adaptations that a human chef might employ. For example, dicing an orange according to the given specification may not lead to an optimal outcome in practice. However, such simplification is a necessary step toward building a generalizable framework, and the present goal is not to prescribe the one best way of performing an action but to demonstrate how structured parameters can enable robots to dynamically execute meal preparation tasks in a systematic and reproducible manner.

### 3.5 Action parameters

As an example, consider this randomly chosen recipe from the dataset for cooking an *Apple And Almond Chutney*:

**Put** the almonds **into** a small bowl and **add in** sufficient boiling water to **cover** them.

**Set aside** for 15 min then **drain** and **put into** a blender.

**Peel** and **core** the apple and **chop** it roughly. **Mix** with the lemon juice and **add in** to the blender together with the remaining ingredients.

**Blend** till smooth.

**Refrigerate** for an hour.

With the defined ACs and AGs we can now translate the verbs of the instructions to parameters of the general action plans of the robot, as exemplarily explained by Beetz et al. (2024) for cutting actions. Extending this idea, the example recipe could thus be translated into the ACs and corresponding parameters shown in Table 4.

**TABLE 4:** Example translation of verbs to action parameters.

Step	Verb	Action core	Action parameter
1	put […] into	pick and place	object, destination
2	add in	mixing	ingredient, destination
3	cover	pick and place	object, destination
4	set aside	pick and place	object, destination
5	drain	pouring	object, sieve
6	put into	pick and place	object, destination
7	peel	preparing	object, tool
8	core	preparing	object, tool
9	chop	cutting	object, tool, position, repetitions
10	mix	mixing	ingredients, motion, tool
11	add in	mixing	ingredient, destination
12	blend	mixing	ingredients, tool, duration
13	refrigerate	cool	destination, duration

### 3.6 Towards preparing any meal

By adding the action parameters of the AGs to the AKG, the meal preparation knowledge graph can be used to parametrise generalised manipulation plans, as demonstrated by Kümpel et al. (2024). However, as a next step towards enabling robots to prepare any meal, the abstract parameters incorporated in the knowledge graph need to be grounded in the actual instruction found in the recipe. In a previous approach by Kanazawa et al. (2024), LLMs are used to extract the concrete values for the available parameters from a natural language instruction. From the instruction *bring a pot of water to a boil*, the LLMs can successfully extract the following instantiation: boil(water, boiled_water). Based on the affirmative results for their use case, we also plan to employ a neuro-symbolic component for the plan parameterization for each concrete recipe to enable robots to perform any necessary meal preparation task in the future.

## 4 Handling unmatched actions

With the direct matching of action verbs in the AGs, we were able to match ∼54% of the verb tokens in the corpus. To improve the coverage, we first calculate the cosine similarity measure between unmatched verbs and the proposed AGs to find similar verbs. With this, we were able to match ∼77% of verbs in the corpus. The remaining, still unmatched verbs are given to LLMs to create new and potentially missing ACs or AGs.

### 4.1 Finding similar verbs and calculating coverage

After analysing the corpus and creating the 6 ACs with their AGs in Section 3, there are still verbs remaining in the dataset that have no connection to our AKGs. To handle these verbs, we calculate the cosine similarity between each unmatched action and all verbs of the six different ACs to find the most similar AG.

**FIGURE 5:**
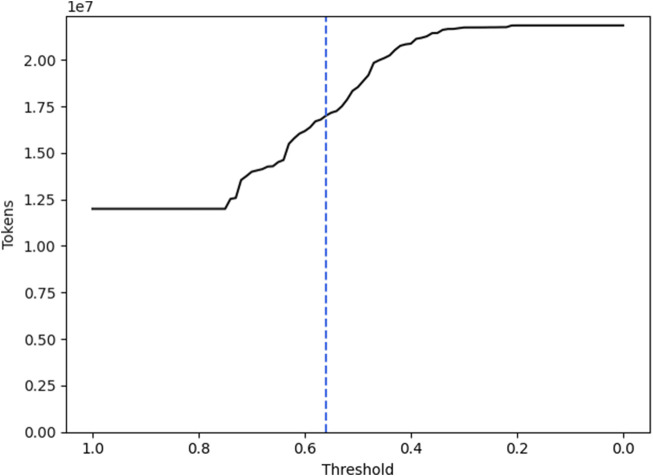
The line shows the total number of verb tokens from the recipe instructions that would be grouped into ACs based on a threshold for their cosine similarity. The dotted line marks the chosen threshold of 0.56.

To determine the threshold of cosine similarity above which unmatched verbs are matched to their most similar AG, we assessed how many verbs were grouped into some existing AC in absolute numbers in Figure 5.

**FIGURE 6:**
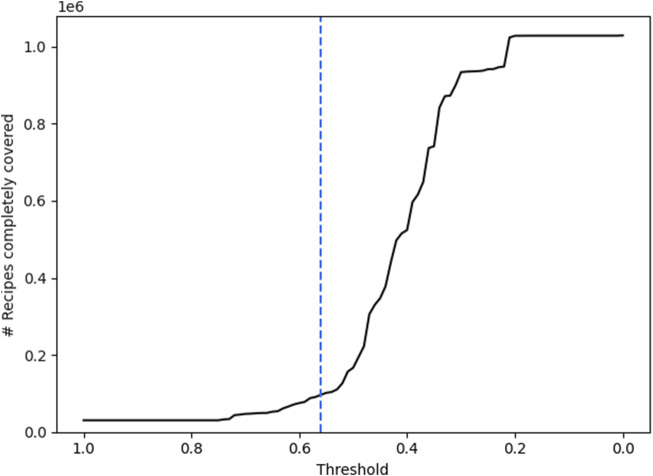
The line shows the amount of recipes whose actions are covered completely when a specific cosine similarity threshold is chosen for grouping the verbs into the ACs. The dotted line marks the chosen threshold of 0.56.

Additionally, we use these resulting matchings and calculate how many instructions are covered in each recipe. The resulting amount of recipes that are covered completely, meaning every action occurring in the recipe is included in the AGs, can be examined in Figure 6.

Four important facts can be drawn from these two graphs:

- There is a large number of verbs that fit into the AGs that are included in the AKGs without any cosine similarity applied. 11,995,642 out of all 22,084,228 verbs (∼54.31%) were matched directly. At the same time, only 30,499 recipes are covered completely (∼2.96%) . Overall, the recipes are covered by ∼54.74% on average.
- At a cosine similarity threshold of 0.01, there are still 583 verbs ungrouped. This amounts to 89 different verb tokens (1.65% of all different verb tokens) and 544 recipes that are not completely covered (0.053% of all recipes).
- The number of verbs that are grouped to one of the ACs is rising at an applied cosine similarity threshold of 0.56. This indicates that the initially chosen clusters have a high distance to the remaining unmatched verbs.
- Down to a cosine-similarity threshold of 0.64 there is a relatively small rise in grouped verbs and completely covered recipes. This indicates that the proposed AGs in the ACs are already covering their respective domain well. If there would be verbs of significant quantity in the corpus that are not included in the ACs but relevant to the domains, there is a high likelihood that they would have been included with a high cosine similarity.

This analysis led us to set the threshold to 0.56. Thereby, 632 distinguished verbs were grouped into one of the 6 ACs and the instructions found in each recipe are covered by 76.51% . We also cover 96,526 recipes completely (9.38%) .

**FIGURE 7:**
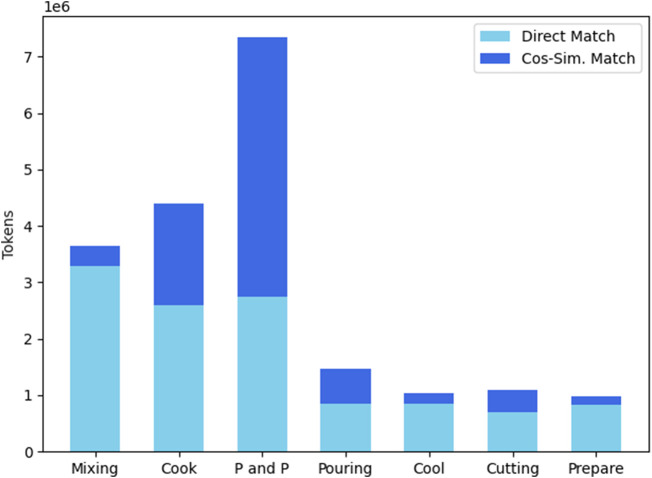
The number of verb tokens that found a direct match in the ACs and the number of verbs that were added to the ACs by allowing a minimum cosine similarity of 0.56. “P and P″ stands for “Pick and Place”.

In Figure 7 the results of matching the verbs from the corpus into the ACs is shown. Moreover, the bars show how many verbs were additionally added to the various ACs by allowing a verb with a minimum cosine similarity of 0.56 to be added to the ACs. What can also be assessed in Figure 7 is the relevance of the different ACs for meal preparation tasks. From the considered ACs, *Cutting* has the lowest presence in the dataset whilst *Mixing* actions have the highest count.

### 4.2 Using Large Language Models to handle further verbs

After matching using the cosine similarity, there are still 4,762 distinguished unmatched verbs that make up roughly 23% of verbs found in the whole recipe corpus. We now want to investigate whether generative LLMs are a suitable source for matching the remaining verbs or coming up with new and currently missing ACs. Since an extensive experiment is beyond the scope of our research, we perform a small feasibility study to investigate the general capabilities and assess whether more research in this direction is advisable. To perform this study, we focus only on a small subset of the remaining unmatched verbs. For this subset we choose the 30 unmatched actions with the most occurrences in the corpus and manually filter them according to two conditions. We exclude:

- Auxiliary verbs (e.g., *make, do, have, let*)
- Abstract verbs that do not describe physical actions (e.g., *remain, need, desire, enjoy*)

After this exclusion, 15 words remain for this pre-study, which we manually mapped to the existing ACs or chose to create a new AC for. These 15 words with their manual mapping are the gold standard for our comparison.

**FIGURE 8:**
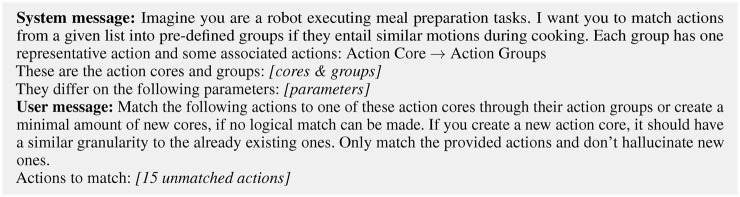
The prompt given to the LLMs for matching 15 of the remaining unmatched actions to the existing cores or creating new cores, if no suitable match is found.

To perform this pre-study, we query OpenAI’s GPT-3.5 and GPT-4o models (OpenAI, 2023), Claude (Anthropic, 2024), Llama 3.3 (Grattafiori et al., 2024) and Gemma 2 (Gemma Team et al., 2024) five times via their respective API using the prompt in Figure 8. For all five runs of the models, the *temperature* is set to zero to create results that are as deterministic as possible.

In our created gold standard, only a single new core was created for the action *repeat*. Of the five models we prompted, only Claude and GPT-4o were able to also propose a similar new core. Of the remaining models, both Llama 3.3 and Gemma 2 showed limited creativity by answering *Nothing* to our matching request, actively working against the request made in the prompt of “creating a minimal amount of new cores, if no logical match can be made” (see Figure 8). This answer was given by Gemma 2 also for two other actions (*wrap* and *store*), which were both matched effortlessly by the other four models. GPT-3.5 did not propose any new cores but tried to match all 15 verbs to the six existing cores. This restrictiveness is in contrast to Claude, which proposed an additional new core (*Coating*) to which it mapped six out of the 15 verbs, and Llama 3.3, which, similar to Claude, proposed the novel action core *Spreading*, to which four actions were matched. Apart from the three actions being unmatched, Gemma 2 does not propose any new cores and GPT-4o does not propose any other cores than the one expected in the gold standard.

Regarding the performance for actually matching the remaining 14 actions to one of the six action cores, the models again vary in their results and in the amount of correct matches. A possible misunderstanding regarding the scope and differentiation of the *Pouring* and *Preparing* action cores is indicated by the pattern of mismatched actions observed in Gemma 2, GPT-3.5 and GPT-4o, where multiple actions correctly associated with *Pouring* are mismatched to *Preparing*. For Claude, apart from the aforementioned mismatches due to the newly proposed action core, only a single mismatch occurs and for Llama 3.3, there is no distinctive pattern in the three mismatches that occur. If we take quantitative measures like the F1-score into account, GPT-3.5 slightly outperforms the other models (F1=0.82) , directly followed by GPT-4o (F1=0.78) . Claude and Llama 3.3 perform on a similar level (F1=0.67) , with Gemma 2 performing worst of all five models (F1=0.60) .

From this small feasibility study on LLM-based action matching, we hypothesise that many of the remaining unmatched actions could, based on their motion-based parameterization, be either mapped into our ACs directly or be decomposed into a combination of the action primitives described by our ACs. Investigating this hypothesis further through a more extensive experimentation is part of our future work.

## 5 Plan parameterization as a core mechanism in adaptive robotics

Plan parameterization plays a key role in enabling robots to adjust their behaviour dynamically. Traditional systems rely on fixed instructions, limiting flexibility. In contrast, our method leverages queries to AKGs to refine task execution in real time. This allows the robot to adapt to varying conditions without requiring constant human intervention, supporting more flexible and efficient automation.

To bridge theory and practice, we have developed an interactive website featuring an experiment section with two main features: 1) inferring action parameters for a chosen recipe and 2) simulating adaptive action execution of different meal preparation actions.

### 5.1 Action parameterization

This section presents how plan parameterization supports adaptive behaviour in robotic systems, enabling robots to dynamically translate abstract actions into executable motion plans. The proposed AKGs and ACs have been implemented in a streamlined pipeline. Once the pipeline is executed, the resulting graph provides a ready-to-use knowledge base for robotic meal preparation. This can be tested on our website^5^, where a user can choose a recipe (out of the Recipe1M + dataset) and explore its structured representation to then get 1) a link to the recipe website, 2) natural language recipe preparation instructions, 3) matched action verbs and 4) associated action parameters, if available. The entire process is fully automatic and needs neither human intervention nor does it rely on LLMs but solely queries the implemented AKGs.

### 5.2 Adaptive action execution

A second interface^6^ allows users to test the robot’s action execution through a simulated environment. The robot processes the selected actions - such as cutting, pouring, or mixing - by retrieving the necessary motion parameters from the AKG, showcasing the direct application of our framework.

The website enables transparent experimentation by allowing users to observe how high-level recipe instructions are transformed into robot-executable commands. This hands-on approach highlights the adaptability of our system, which no longer requires executing the full pipeline for each task; once the graph is generated, it can be reused for various recipes and their corresponding actions.

Through this setup, we validate the flexibility and practicality of our approach: the robot dynamically adjusts its execution plans, grounded in the AKG’s structured knowledge, while providing a user-accessible tool for both research and educational purposes.

**FIGURE 9:**
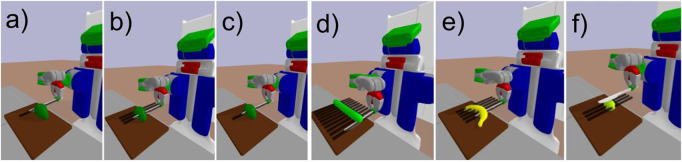
The PR2 robot demonstrates cutting techniques on various fruits: **(a)** cutting, **(b)** slicing, **(c)** halving an avocado, slicing **(d)** a cucumber, **(e)** a banana or **(f)** a lemon. The black lines represent motion trajectories.

Figure 9 demonstrates the robot’s proficiency in adjusting its execution plan for tasks like halving and slicing without prior knowledge of the objects, relying solely on information retrieved from the AKG during execution. The cutting scenario can be tested with all verbs available in the ACs, and on a range of objects, as also detailed in (Kümpel et al., 2024). Our findings underline the robot’s adaptability across a range of scenarios, setting a foundation for further advancements. Additionally, our entire setup is available online and accessible to individuals^7^.

This example serves as a starting point for examining more complex capabilities. By isolating the key process of plan parameterization, we set the stage for a deeper exploration of the system’s computational architecture, especially by investigating the uncertainties in perception and execution that we abstracted away from. This structured approach underscores the importance of plan parameterization in creating adaptable robotics while connecting the concept to broader advancements in system design and decision-making.

### 5.3 Limitations

While the experiments demonstrate that the proposed Actionable Knowledge Graphs provide sufficient parameters for executing diverse cooking actions, several limitations remain. First, the current parameterization is deliberately simplified, which enables systematic robot execution but may not capture the full variability of human cooking strategies or yield the most efficient motions in every context. Second, the approach relies on robust perception and environment representations, yet real kitchen scenarios often involve uncertainty, occlusion, and noisy object recognition. Third, although the method covers approximately 77% of verbs in the corpus, a notable portion of less frequent or complex actions remains unmatched, requiring further work on expanding and refining the AGs. Finally, the validation is limited to simulated execution and selected robot demonstrations; more extensive physical experiments are needed to assess robustness in real-world kitchen environments.

Beyond these methodological issues, several practical limitations also remain. The current AGs do not model actions in relations to cooking processes, nor their prioritisation—e.g., whether waiting for a cake to bake should take precedence over performing a new action—are not yet represented in the AKGs. Addressing such autonomy concerns will be crucial for bridging the gap between experimental validation and real-life deployment, as well as going from execution of single actions to meal preparation. These limitations do not undermine the value of our approach as a conceptual and technical proof of concept but rather point to future research directions: testing the framework in long-horizon, open-ended meal preparation scenarios with real robots.

## 6 Conclusion

Towards the goal of empowering robots to successfully prepare varying meals, in this paper we introduce six Action Cores that were identified as central manipulation action categories in the analysed recipe corpus. For each AC we include Action Groups that summarise all actions that result in similar motion parameters and similar manipulation outputs. We match the action verbs found in the Recipe1M + corpus to our ACs and AGs in two steps: First, we match them directly, covering ∼54% of all verbs. Afterwards, we match the remaining verbs using cosine similarity and an experimentally defined threshold, leading to a coverage of ∼77% .For the remaining verbs, we query LLMs to match the verbs or provide us with additional ACs that possibly cover the missing verbs, but a brief analysis shows that the newly proposed cores are already incorporated by our proposed cores, underlining their great coverage for meal preparation tasks.

The presented approach is limited mostly by the initial choice of action cores as well as their associated action groups. As explained throughout this paper, we use our analysis to underline the relevance of the created ACs and AGs, but a slight change in this initial setup would hinder the repeatability of the direct matching, the similarity-based matching as well as the neuro-symbolic experiment. Additionally, the proposed approach focuses on the task of matching verbs to the ACs for incorporation into the knowledge graph. However, the matching alone is no guarantee for a successful execution, as the knowledge needs to be correctly grounded in the action-perception-loop of the robot, an aspect we want to investigate in future work.

In the future, we want to take the next step towards robots automatically preparing any meal they encounter by including a neuro-symbolic component that extracts the natural language parameters from the actual recipe text to create the concrete parameterization of each action, as we explained in Section 3.6. Additionally, we want to perform more robotic experiments to investigate the adaptability of the proposed approach as well as the practicability of the ACs. Lastly, we need to investigate further how the remaining unmatched words can be handled and whether they can be, e.g., automatically decomposed into sequences of existing ACs or AGs, as hypothesised in Section 4.2.

## Data Availability

The Recipe1M+; dataset used in this study can be found on this website: https://im2recipe.csail.mit.edu/. An ontology subset of this dataset is available on this website: https://michaelakuempel.github.io/ProductKG/Ontologies.html. The software written to analyse the recipe data is publicly available on GitLab: https://gitlab.ub.uni-bielefeld.de/mscheibl/recipeanalyser. All experiments are publicly available on the linked websites.

## Footnotes

1 The experiment is openly available online: https://vib.ai.uni-bremen.de/page/labs/action-cores/

2 Test the idea online: https://vib.ai.uni-bremen.de/page/labs/from-recipes-to-actions/

3 A subset of the recipe dataset in OWL format can be found here: https://michaelakuempel.github.io/ProductKG/Ontologies.html

4
https://spacy.io/models/en

5 The action parameterization website can be accessed here: https://vib.ai.uni-bremen.de/page/labs/from-recipes-to-actions/

6 The robot simulation for adaptive action execution is available at https://vib.ai.uni-bremen.de/page/labs/action-cores/

7 The hands-on code is available at https://vib.ai.uni-bremen.de/page/labs/actionable-knowledge-graph-laboratory/
